# No Child Should Be Left Behind by COVID-19: A Report about the COVID-19 Pandemic Experience in Children and Adolescents with Acute or Chronic Disease Treated at a Pediatric Referral Hospital in Italy

**DOI:** 10.3390/pediatric15010008

**Published:** 2023-01-28

**Authors:** Giulia Zucchetti, Sabrina Ciappina, Cristiana Risso, Alice Malabaila, Sara Racalbuto, Elena Longo, Margherita Dionisi Vici, Marina Bertolotti, Paola Quarello, Franca Fagioli

**Affiliations:** 1 Pediatric Oncohematology Division, Regina Margherita Children’s Hospital, Azienda Ospedaliera-Universitaria Città della Salute e della Scienza, University of Torino, 10126 Torino, Italy; 2 Pediatric Pneumology Division, Regina Margherita Children’s Hospital, Azienda Ospedaliera-Universitaria Città della Salute e della Scienza, 10126 Torino, Italy; 3 Pediatric Emergency Division, Regina Margherita Children’s Hospital, Azienda Ospedaliera-Universitaria Città della Salute e della Scienza, 10126 Torino, Italy; 4 Child and Adolescent Neuropsychiatry Division, Regina Margherita Children’s Hospital, Azienda Ospedaliera-Universitaria Città della Salute e della Scienza, 10126 Torino, Italy

**Keywords:** COVID-19, acute and chronic disease, pediatric patients, hospital, Italy

## Abstract

Background: The pandemic of coronavirus disease 2019 (COVID-19) was undoubtedly a stressful experience for everyone. General opinion believed that children with acute or chronic illness could experience additional burden, but this is not confirmed. The aim of this study is to understand how children and adolescents already suffering from acute or chronic illness (e.g., cancer, cystic fibrosis, neuropsychiatric disorders) feel about the COVID-19 pandemic, and if the experience is significantly different between these children and children without illness. Methods: Children and adolescents affected by acute or chronic illness (named the “fragile group”) treated at the Regina Margherita Children Hospital in Italy, were enrolled in the study by filling a questionnaire about their pandemic experiences. Also, a group of children and adolescents without acute or chronic illness (named the “low-risk group”) recruited in the emergency department of the hospital, participated in the study in order to compare experiences. Results: The study group was composed of 166 children and adolescents (Median age = 12 yrs; 78% fragile group, 22% low-risk group). Participants experienced a general state of fear of the virus and of a potential infection for both themselves and their families, while feelings and thoughts that interfere with daily functioning were less frequent. The fragile group seems to be more resilient towards the pandemic situation than low-risk group and some differences on the basis of the type of illness were found in the fragile group. Conclusions: Dedicated psychosocial intervention must be proposed in order to support fragile children and adolescents’ well-being during the pandemic, also on the basis of their clinical and mental history.

## 1. Introduction

Since February 2020, Italy has been one of the countries strongly affected by Coronavirus disease 19 (COVID-19). Most of the COVID-19 cases were registered in Northern Italy: in Piedmont, until November 2020, there were more than 900 000 cases (www.salute.gov.it, accessed on 10 July 2022) registered. Although the incidence of positive cases among children (0–18 years) is only around 1–2% it is well known that children and adolescents already affected by a chronic or acute illness are at major risk compared to their peers [1]. Some pediatric conditions, such as children with cancer (the main cause of death in infancy) or cystic fibrosis (the most common lethal disease in the Caucasian population), are likely to be more vulnerable to infections due to their immunological and pulmonary situation [2,3,4].

In addition to the well-known physical effects, COVID-19 also implies severe acute and long-term adverse mental health outcomes for children and adolescents in general. It is well recognized that in the pediatric population, most feelings towards the virus include fear, loneliness, anxiety, and depression, especially due to the social isolation [5,6]. Also, adolescents may feel frustrated, nervous, nostalgic, and bored because of social distancing [7]. Some studies show difficulty in sleeping [8] and a higher risk of depression and anxiety [9].

Although these psychological outcomes of the pandemic are recognized among children and adolescents in general, among children and adolescents with previous diseases, little is known about their emotional experience.

The literature mainly presents studies relating the emotional impact on parents of children with previous disease. The majority of parents were worried about COVID-19 and transmitting the virus to their child [10].

Also, one study reports that parents of pediatric cancer patients, during the pandemic, have a high psychological risk for post-traumatic symptoms, high stress levels, and the presence of clinically significant levels of anxiety [11].

For children and adolescents with previous illness, it could be hypothesized that, given their fragility in physical terms, they may also present an emotional fragility in the face of particular situations such as the pandemic, or on the other hand, it could be hypothesized that such fragility can be a resource for them in dealing with difficulties, but to our knowledge there are no studies that support these hypotheses. A study was found that revealed that COVID-19 had an impact on the mental and physical well-being of youth worldwide, particularly among those with chronic health conditions [4]. For these types of patients it is necessary to have an increased awareness of the likely challenges and opportunities faced by this group and an integrated approach to their care is likely to optimize their psychosocial well-being [4].

At the Regina Margherita Children’s Hospital, the pediatric referral hospital in the Piedmont Region, from the first pandemic wave, dedicated pathways have been produced for patients and their caregivers. Areas were organized to evaluate suspected or confirmed COVID-19 cases and only urgent surgical and outpatient activities were planned. In addition, guidelines for visitors were issued, such as restrictions on visiting and suspension of educational and recreational activities [10,12,13]. All of these rules have added more difficulties in terms of family organization and general burden on already stressed caregivers.

Pediatric clinicians have adapted treatment and care protocols to the new circumstances. Psychological care and recreational activities were changed by moving them to a remote modality (healthcare technology, telephone, etc.) to also give constant support for caregivers at home and to reduce social isolation which was even more severe due to the pandemic [9].

Maintaining attention to patient care, to avoid excessive isolation was found to be essential to promote coping and resilience strategies among patients and caregivers who, in addition to the disease, found themselves fighting the fear of contracting the virus. Also, in order to contain the excess fear, it was very important to assist patients in selecting the most reliable information on the pandemic situation, such as avoiding ‘fake news’ from the internet [14].

In order to propose effective and timely solutions to the various pandemic outcomes, a mapping of the emotional experience of children and adolescents admitted to the Regina Margherita Children’s Hospital in Turin, Italy was carried out. These patients suffer from complex and potentially fatal diseases, which require timely diagnosis and prolonged treatment. They are, therefore, patients who regularly attend the hospital and experience physical and social limitations outside of those induced by the pandemic. The possibility of having a large sample, representative of fragile pediatric patients, permits the researchers to highlight any differences among patients with organic and mental illness and also, based on their phase of treatment. Also, emotional experience and feelings of diseased children and adolescents can be compared to those of children and adolescents without previous illness.

Therefore, the research question of the study is—what is the COVID-19 experience of children and adolescents affected by acute or chronic illness (e.g., cancer, cystic fibrosis, neuropsychiatric disorders) compared to the experience of children and adolescents without disease? Results will permit identification of patients at greatest risk and thus prevent possible long-term adverse effects through the proposal of dedicated interventions drawn up within a customized protocol.

## 2. Material and Methods

### 2.1. Participants and Groups

The research adopted a cross-sectional methodology based on the administration of a questionnaire to children and adolescents treated at the Regina Margherita Children’s Hospital (Turin, Italy). We enrolled children from 6 years old, because they were considered able, from a cognitive and emotional point of view, to understand the purpose of the study, up to 15 years because for some departments this is the age limit for admission to the hospital. Based on our clinical experience it was planned to divide the target sample into two groups: the “fragile group” was composed of children and adolescents in ordinary and daycare hospitalization at the hospital with acute and/or chronic diseases. By ‘fragile group’, we mean those categories of patients suffering from organic diseases such as cancer, cystic fibrosis and other organ failure diseases, and mental diseases such as neuropsychiatric disorders and abuse and neglect diseases. The “low-risk group” was instead composed of children and adolescents who were admitted to the emergency department of the hospital with a ‘white code’, which for the Italian health system are those patients who are not considered urgent/emergency cases and who are not suffering from acute and/or chronic diseases. Green code patients, which refers to minor emergencies, were excluded because the condition of a disturbance due to symptoms or injuries could potentially interfere with the response to the questionnaire. These groups of patients were representative of the main pediatric severe disease patients and allowed us to have good validity in our study.

Participants were enrolled during the second pandemic wave (from Autumn 2020 to Spring 2021). Written informed consent was obtained from patients and parents after explaining the study.

### 2.2. Questionnaire

After an extensive review of the literature concerning pediatric patients and COVID-19, a voluntary anonymous and targeted questionnaire has been developed.

Studies about the impact of COVID-19 on the adult population [14,15] and about the pediatric population [16] were found in literature. Based on these studies, some items were chosen and adopted by a multidisciplinary group of experts in pediatrics, in order to collect the data: the questions can be easily understood and the best conditions for the collection of a large number of answers was created.

Emotional experience and feelings were investigated through four simple but precise items which represented possible areas of the COVID-19 experience for pediatric patients (fear of catching the coronavirus, fright from news on television or media, fear of contagion for oneself and parents, change of life due to coronavirus).

The questionnaire also investigated the socio-demographic and clinical data of the patients involved in the study. The patients were invited to complete the questionnaire during clinical visits or recreational activities proposed by the hospital. A psychologist gave each patient a printed copy of the questionnaire in person and explained the aims of the study. The questionnaire was administered in an anonymous format and completed by patients in their own time. If necessary, assistance or supervision on the part of a psychologist was given; thus, ensuring the confidentiality of all the data collected.

### 2.3. Statistical Analysis

Percentage values were computed to analyze the distribution of answers for each item. Chi-square analysis was performed to investigate possible associations between answers and types of variables investigated (type of disease, phase of treatment, age and gender, fragile and low-risk group). Significant associations were considered under the *p* value of 0.05; R software (version 3.4.1) was used to perform the statistical analysis.

The study was approved by the Ethics Committee with protocol number N° 0060378. Data were collected on an Excel spreadsheet for analysis.

## 3. Results

166 patients were enrolled in the study, with an average age of 12 years (Standard deviation 3.7), 52% were female (n = 86) and 48% were male (n = 80). The most critical issues due to COVID-19 were job loss (4%) and significant bereavement (7%) experienced by patients and their families. The fragile group accounted for 78% (n = 129). In this group, 34% (n = 57) patients suffer from cancer, 16% (n = 26) from cystic fibrosis, 17% (n = 27) from mental and sexual abuse, and 11% (n = 19) from psychiatric disease. A total of 58% (n = 75) of patients were in a course of treatment and 42% (n = 54) had finished the active treatment. A total of 22% (n = 37) of the entire sample were in the low-risk group recruited from emergency room. All the characteristics of the sample are presented in Table 1.

Table 2 shows that females are afraid of contracting the virus themselves (29%) (χ = 10,17 *p* < 0.05), and they are frightened of their parents becoming infected (35%) (χ = 3,1 *p* = 0.21). They also claim that their life has changed for the worse (74%) (χ = 0,96 *p* = 0.32). The difference in residence, highlights that those who live in the city have suffered the most from the coronavirus, especially in terms of quality of life: 85% of patients who live in town declare that their life changed for the worse (χ = 7,74 *p* < 0.05), (Table 2).

Difference by age highlights that older participants are less afraid to catch the virus: 18% of patients >12 years report to have feared less frequently (18%) than younger patients: 61% of patients >12 years declare that they were rarely afraid of contracting the virus (χ = 3,34 *p* = 0.18).

Within the fragile group (Table 3), with respect to the difference between patients with organic and mental illness, we found more fear of catching the coronavirus among children and adolescents with organic illness (21%) than for those with mental illness that report never having been afraid of catching the virus (62%) (χ = 1,34 *p* = 0.51).

Nonetheless, children and adolescents with mental illness report that their life has changed for the worse (94%), while for patients with an organic disease (37%), life seemed to have even improved (χ = 11,97 *p* < 0.05).

Regarding the phase of therapy, no active treatment group seemed to have more frequency of fear of getting the coronavirus (23%), compared to patients in active treatment (18%) (χ = 0,76 *p* = 0.68). Patients who have finished the treatment declared that their life changed for the worse due to coronavirus (72%) (χ = 0,18 *p* = 0.67) (Table 4).

Considering the difference between the fragile group and the low-risk group, results are expressed in Table 5. A total of 24% of the low-risk group have often been afraid of catching the virus, while 57% of the fragile group declared that they have never been afraid of contracting the virus (χ = 1,58 *p* = 0.45), and 71% report that they never got scared hearing about the coronavirus from the media (χ = 2,59 *p* = 0.27). The situation between the fragile (28%) and the low-risk group (32%) is similar with respect to the fear that family members could catch the coronavirus (χ = 0,59 *p* = 0.74).

A total of 32% of the fragile group declared that their life has changed for the better due to the coronavirus, while for the low-risk group, life has changed for the worse (81%) (χ = 2,3 *p* = 0.13).

## 4. Discussion

This study explored feelings about COVID-19 of children and adolescents with acute or chronic illness by examining their experiences through a self-report questionnaire.

COVID-19 has great emotional impact, especially among the pediatric population [5,6]. However, little is known about emotional experience among children and adolescents with specific medical conditions.

Given the uniqueness of the pediatric population, a tailored questionnaire able to report different dimensions of patient’s experience of COVID-19 was created. Pediatric patients represent a slice of patients unique for their delicate phase of life, in mutation and continuous growth, and which can include very different ages, from infancy to adolescence [3]. They also face illness and hospitalization together with their parents or caregivers; therefore their emotions and perceptions are often influenced by the presence of their reference adults. For this reason, it is interesting to observe their responses to the impact of an extraordinary event such as COVID-19 inside a children’s hospital.

Participants reported fear of coronavirus, in particular relating to the possibility of being infected, or that a family member may be infected.

Although studies have shown few cases of child patients infected by COVID-19, even in countries such as Italy where the virus was widespread, for example among cancer patients [17], the fear of contagion is still present in children and adolescents [4]. In general, it seems that females are more frightened by the possibility of contagion than males. This result could be linked to the hypothesis of the gender differences in stress response, but it needs further investigation to be confirmed. Also, it seems that out-of-town participants coped less severely with the pandemic. This result is in line with the fact that the pandemic is making people reconsider city living, by preferring less populated areas.

In our sample, this fear does not appear to be due to the news that the participants received from the media. Moreover, this fear does not seem to generate too much discomfort in thinking about the virus and does not seem to affect patients’ daily life, especially their sleeping areas.

Our results, combined with clinical practice and observation, showed that pediatric patients had understood the pandemic situation and the potential adverse outcomes of coronavirus, involving a general fear for oneself and others.

These observations agree with those shown in a study from Saudi Arabia, demonstrating that most ill children interviewed were fearful of themselves or a family member contracting the virus [18]. For example, cancer patients contracting the virus could mean having to stop treatment, a worsening health condition and in some cases having a worse prognosis. These patients are afraid to catch the virus, but they are also the ones most used to protecting themselves and being careful not to get infections. With the virus, therefore, their protection methods have not changed much. For this reason, the life of organic disease patients does not seem to have worsened, indeed in some ways it has also improved, probably because certain protections no longer concern them only, but must be adopted by all their peers. This is not the case for patients with mental illness as they are not physically vulnerable or at greater infectious risk, they have had to change their habits a lot and have probably experienced a deterioration in their quality of life. That is a fact that seems to be confirmed by our results.

Some studies in fact have highlighted the important burden of COVID-19 on adolescent mental health especially among those with pre-existing problems: requests for psychological support, mental problems and above all eating disorders have increased [19].

Considering the clinical phase of participants, our results did not show many differences between patients in active treatment and those who had finished treatment. However, patients who had finished treatment appear to express a greater general fear of the virus than patients in active treatment. This evidence could be related to the anxiety and distress that survivors often report. For cancer survivor patients for example, they are at risk of complications due to their past cancer treatments and at risk for a serious course of COVID-19 infection [20]. The general fear of the virus by childhood cancer survivors can be due to adopting behaviors (e.g., self- isolation) and to the health recommendations to remain vigilant about symptoms that can remind them of the past cancer treatment period [21].

Patients in active treatment may focus their energy in healing the disease and not on the COVID-19, making them look more resilient than survivors. Furthermore, some research shows how the restrictions due to virus have helped patients in active treatment to feel less alone and to encourage their peers in coping with restrictions and social isolation [22,23].

Finally, our study allowed us to compare the pandemic experience of patients and children and adolescents without previous illness. Based on clinical experience, groups of pediatric patients were named as the “fragile group” given their objective difficulties due to their disease, while children and adolescents without previous illness were placed in the “low-risk group”. However, to our knowledge no information about the experience of the two groups was found in literature. Our descriptive analysis underlined an interesting difference between the fragile and low-risk group. The first one appears less scared by the virus. The low-risk group, represented by children or adolescents without significant disease reports being afraid to catch the virus and to be scared by the media. They also report that their life changed for the worse due to the virus. This is probably because patients without acute or chronic diseases are not used to social isolation and to paying more attention to their health than their peers.

Certainly, COVID-19 and its containment measures have had a strong emotional impact on the mental health and daily habits of both low-risk children and those with pre-existing chronic diseases. However, given the unicity of the pediatric population, their experience of COVID-19 should be deepened.

This study showed that our participants have a general fear of COVID-19 and its consequences, but at the same time they also manifest a resilience capacity: most of them indeed, do not report great difficulties in daily life (e.g., sleeping).

This adaptation can be explained by the continuity and by the increase in psychological support that pediatric patients receive. Furthermore, reduced academic and social stressors, increased time with families, reduced access to substances, easier access to health care using technology, and opportunities to build resilience may also have been experienced by pediatric patients as a psycho-social opportunity [24,25]. Nonetheless, it is very important to remember as pointed out by Sara Mitchell [26], that children with medical complexity faced unique challenges in the current pandemic and the healthcare providers must improve the care delivery for both the child and his or her family. It is on this type of pediatric population, the fragile ones, that future research must focus to provide for them targeted support interventions. Also, good communication and information about COVID-19 suitable for children and adolescents are essential to avoid unanswered doubts and to contain fears.

## 5. Study Limitations

Information from parents on the psychosocial well-being and quality of life of their children may further enhance our understanding of the impact of the pathology and its treatments on the children. Also, this is a descriptive study, so the results presented in the current research must be confirmed and deepened in subsequent studies by adopting a larger sample. Finally, the low-risk group is undoubtedly less represented, allowing us only a descriptive comparison.

## 6. Clinical Implications

Children with acute and chronic illness, despite their true resilience, have a special sensitivity, which can make them experience the COVID-19 pandemic in a particular way.

So, multidisciplinary mental health teams (psychiatrists, psychiatric nurses, clinical psychologists) together with pediatricians, social workers and non-governmental organizations should be involved in providing targeted interventions in vulnerable children.

Our study has allowed us to highlight the conditions of most fragile patients in the face of a health emergency and to propose for them dedicated strategies so they do not fall further behind and can have the same opportunities as their peers.

This may help should similar emergencies arise in the future. In fact, it has allowed us to understand the importance of not leaving any child behind in the pandemic and to propose dedicated psychosocial interventions in order to support the well-being of children and adolescents also on the basis of their clinical and mental history.

## Figures and Tables

**Table 1:** Characteristics of the sample.

Whole Sample	166
Gender	
*Female*	86 (52%)
*Male*	80 (48%)
Age	
*<12 years*	74 (45%)
*>12 years*	92 (55%)
*Mean, Standard deviation, age range*	12 (SD 3.7, range 6–15)
Place of residence	
*City*	50 (30%)
*Out of city*	116 (70%)
COVID-19 issues	
*Job loss*	7 (4%)
*Significant bereavement*	12 (7%)
**Fragile Group**	**129 (78%)**
Organic diseases	
*Cancer*	57 (34%)
*Cystic Fibrosis*	26 (16%)
Mental diseases	
*Mental and sexual abuse*	27 (17%)
*Psychiatric diseases*	19 (11%)
Phase of treatment	
*No active treatment*	54 (42%)
*Active treatment*	75 (58%)
Age	
*Mean, Standard deviation, age range*	13.01 (SD 4, range 6–15)
**Low-Risk Group**	**37 (22%)**
Age	
*Mean, Standard deviation, age range*	12.08 (SD 3.8, range 6–15)

**Table 2:** Emotional experience and feelings towards the pandemic considering gender, residence and age of the whole sample.

		Always	Sometimes	Never
1. I was afraid of catching the coronavirus	Female	29% (N = 25)	26% (N = 22)	45% (N = 39)
	Male	10% (N = 8)	26% (N = 21)	64% (N = 51)
	City of Turin	22% (N = 11)	24% (N = 12)	54% (N = 27)
	Out of town	19% (N = 22)	27% (N = 31)	54% (N = 63)
	<12 years	22% (N = 16)	30% (N = 23)	48% (N = 35)
	>12 years	18% (N = 17)	21% (N = 19)	61% (N = 56)
2. I have heard of coronavirus on television and got scared	Female	17% (N = 15)	26% (N = 22)	57% (N = 49)
	Male	5% (N = 4)	14% (N = 11)	81% (N = 65)
	City of Turin	14% (N = 7)	26% (N = 13)	60% (N = 30)
	Out of town	11% (N = 13)	17% (N = 20)	72% (N = 83)
	<12 years	14% (N = 10)	19% (N = 14)	67% (N = 50)
	>12 years	8% (N = 7)	21% (N = 20)	71% (N = 65)
3. I was afraid that my parents or friends might catch the coronavirus	Female	35% (N = 30)	36% (N = 31)	29% (N = 25)
	Male	22% (N = 18)	44% (N = 35)	34% (N = 27)
	City of Turin	30% (N = 15)	38% (N = 19)	32% (N = 16)
	Out of town	28% (N = 32)	41% (N = 48)	31% (N = 36)
	<12 years	32% (N = 24)	36% (N = 26)	32% (N = 24)
	>12 years	25% (N = 23)	44% (N = 41)	31% (N = 28)
		**Better**	**Worse**	
4. Has your life changed for the better or worse since the coronavirus?	Female	26% (N = 22)	74% (N = 64)	
	Male	33% (N = 26)	67% (N = 54)	
	City of Turin	15% (N = 7)	85% (N = 43)	
	Out of town	35% (N = 41)	65% (N = 75)	
	<12 years	30% (N = 22)	70% (N = 52)	
	>12 years	29% (N = 27)	71% (N = 65)	

**Table 3:** Emotional experience and feelings towards the pandemic within the fragile group (organic vs. mental diseases) (n = 129).

		Always	Sometimes	Never
1. I was afraid of catching the coronavirus	Organic diseases	21% (N = 17)	25%(N = 21)	54% (N = 45)
	Mental diseases	14% (N = 6)	24% (N = 11)	62% (N = 29)
2. I have heard of coronavirus on television and got scared	Organic diseases	12% (N = 10)	15% (N = 12)	73% (N = 61)
	Mental diseases	5% (N = 2)	38% (N = 18)	57% (N = 26)
3. I was afraid that my parents or friends might catch the coronavirus	Organic diseases	25% (N = 21)	40% (N = 33)	35% (N = 29)
	Mental diseases	43% (N = 20)	43% (N = 20)	14% (N = 6)
		**Better**	**Worse**	
4. Has your life changed for the better or worse since the coronavirus?	Organic diseases	37% (N = 31)	63% (N = 62)	
	Mental diseases	6% (N = 3)	94% (N = 43)	

**Table 4:** Emotional experience and feelings towards pandemic within fragile group (active and no active treatment) (n = 129).

		Always	Sometimes	Never
1. I was afraid of catching the coronavirus	In therapy	18% (N = 13)	25%(N = 19)	57% (N = 43)
	Off therapy	23% (N = 12)	27% (N = 15)	50% (N = 27)
2. I have heard of coronavirus on television and got scared	In therapy	11% (N = 8)	23% (N = 17)	66% (N = 50)
	Off therapy	11% (N = 6)	16% (N = 9)	73% (N = 39)
3. I was afraid that my parents or friends might catch the coronavirus	In therapy	27% (N = 20)	40% (N = 30)	33% (N = 25)
	Off therapy	31% (N = 17)	40% (N = 22)	29% (N = 15)
		**Better**	**Worse**	
4. Has your life changed for the better or worse since the coronavirus?	In therapy	28% (N = 21)	72% (N = 54)	
	Off therapy	31% (N = 17)	69% (N = 37)	

**Table 5:** Emotional experience and feelings towards pandemic between the fragile group and the low-risk group.

		Always	Sometimes	Never
1. I was afraid of catching the coronavirus	Fragile group	18% (N = 23)	25% (N = 32)	57% (N = 74)
	Low-risk group	24% (N = 9)	30% (N = 11)	46% (N = 17)
2. I have heard of coronavirus on television and got scared	Fragile group	10% (N = 13)	19% (N = 24)	71% (N = 92)
	Low-risk group	19% (N = 7)	22% (N = 8)	59% (N = 22)
3. I was afraid that my parents or friends might catch the coronavirus	Fragile group	28% (N = 33)	40% (N = 53)	32% (N = 41)
	Low-risk group	32% (N = 12)	38% (N = 14)	30% (N = 11)
		**Better**	**Worse**	
4. Has your life changed for the better or worse since the coronavirus?	Fragile group	32% (N = 41)	68% (N = 88)	
	Low-risk group	19% (N = 7)	81% (N = 30)	

## Data Availability

Data were collected on an Excel spreadsheet for analysis.
